# Bioinformatics Analysis and Experimental Validation of ASF1B in Breast Tumors

**DOI:** 10.1002/cam4.71073

**Published:** 2025-09-13

**Authors:** Wenhao Xing, Meng Deng, Wendong Wang, Yueqi Liu, Xuefang Mi, Huixia Li, Xin Ge

**Affiliations:** ^1^ Department of Breast Surgery The First Affiliated Hospital of Zhengzhou University Zhengzhou China

**Keywords:** anti‐silencing function protein 1B, breast cancer, clinical prognosis, immune infiltration

## Abstract

**Objective:**

To investigate the association between ASF1B expression and pathological characteristics of breast cancer, and to further explore its role in tumor progression and the immune microenvironment, thereby evaluating its potential as a therapeutic target.

**Methods:**

ASF1B expression in breast cancer was analyzed using the GEPIA2 and BEST databases. Its association with patient prognosis was assessed using Kaplan–Meier survival analysis. Protein co‐expression networks were constructed using GeneMANIA. The correlation between ASF1B expression and immune cell infiltration was evaluated through the TIMER platform. Experimental validation was performed using qPCR and immunohistochemistry (IHC) on 67 breast cancer tissue samples.

**Results:**

ASF1B expression was significantly elevated in breast cancer tissues compared to normal tissues (*p* < 0.05). High ASF1B expression was associated with reduced overall and recurrence‐free survival (*p* < 0.05). Protein interaction analysis revealed that ASF1B was strongly linked to proteins involved in DNA replication, cell cycle progression, and chromatin remodeling. Immune analysis indicated positive correlations with B cells, neutrophils, and dendritic cells and a negative correlation with macrophage infiltration (*p* < 0.05). Clinical data further showed that high ASF1B expression was significantly associated with HER2‐positive breast cancer (*p* = 0.026).

**Conclusion:**

ASF1B is highly expressed in breast cancer and correlates with poor prognosis and immune cell infiltration. It may serve as a potential prognostic biomarker and therapeutic target in breast cancer.

## 1 Introduction

Breast cancer is a heterogeneous disease comprising multiple molecular subtypes, each characterized by distinct genetic, phenotypic, and clinical profiles. Despite advances in treatment strategies—including surgery, radiotherapy, chemotherapy, targeted therapy, and immunotherapy [1]—prognosis remains poor in certain subgroups such as HER2‐positive and triple‐negative breast cancer (TNBC). This underscores the urgent need to identify new biomarkers and therapeutic targets that reflect the complexity of breast cancer biology [2, 3].

Anti‐silencing function protein 1B (ASF1B), a histone H3‐H4 chaperone, plays a critical role in DNA replication, chromatin assembly, and cell cycle regulation [4]. Unlike its isoform ASF1A, which is more involved in transcriptional regulation and senescence, ASF1B is predominantly associated with cell proliferation and tumor progression. Previous studies have identified ASF1B as a potential oncogene in several malignancies, including lung, gastric, and liver cancers [5, 6, 7, 8, 9, 10, 11, 12, 13, 14]. However, its role in breast cancer, especially in relation to molecular subtypes and immune modulation, remains poorly understood.

Given the central role of ASF1B in chromatin dynamics and proliferation, we hypothesized that ASF1B expression may be elevated in breast cancer tissues and associated with adverse clinicopathologic features and prognosis. Moreover, we proposed that ASF1B may influence the tumor immune microenvironment, thereby modulating immune cell infiltration patterns. To test these hypotheses, we performed an integrative analysis combining bioinformatics interrogation of public databases with validation in clinical breast tumor samples. This study aims to define the expression pattern of ASF1B in breast cancer, assess its prognostic significance, explore its association with immune infiltration, and lay the groundwork for future translational research.

## 2 Materials and Methods

### 2.1 Bioinformatics Analysis

RNA sequencing data and clinical information were obtained from the UCSC Xena Browser (https://xenabrowser.net) [15]. Gene expression differences between normal and tumor tissues were analyzed using RStudio (v4.4.3). The GEPIA2 platform was used to compare ASF1B expression in breast cancer and normal tissues [16]. Kaplan–Meier survival analysis was performed using KMplot (http://www.kmplot.com) [17]. The TIMER database assessed correlations between ASF1B expression and immune cell infiltration [18]. The GeneMANIA platform was used to construct ASF1B‐related protein interaction networks [19]. Expression analysis in multiple GEO datasets and enrichment analyses (GO and KEGG) were conducted using the BEST tool (https://rookieutopia.hiplot.com.cn/app_direct/BEST/).

All public datasets were used with default normalization and quality control settings provided by each platform. Data from multiple databases were cross‐validated to ensure consistency of ASF1B expression patterns.

### 2.2 Clinical Samples

A total of 67 paired breast tumor and adjacent non‐cancerous tissue samples were collected from patients who underwent surgery at the First Affiliated Hospital of Zhengzhou University. Inclusion criteria: (1) histologically confirmed breast cancer, (2) no neoadjuvant therapy, and (3) complete clinical information. Exclusion criteria: (1) history of other malignancies, (2) prior chemotherapy or radiotherapy, or (3) incomplete sample data. All participants signed informed consent, and the study was approved by the hospital's Ethics Committee (Approval No. 2024‐KY‐2094‐002).

### 2.3 Total RNA Extraction and qPCR

Total RNA was extracted from 23 tumor and paired adjacent tissues using TRIzol reagent (Thermo Fisher). Reverse transcription was performed using PrimeScript RT Master Mix (Takara). Quantitative PCR (qPCR) was carried out using SYBR Green Master Mix (Applied Biosystems). Primer sequences used were: ASF1B: Forward: 5′‐GATGGTATCAGAGGGCCTGC‐3′, Reverse: 5′‐GGGAGGGACAGTTTTCAGGG‐3′ GAPDH: Forward: 5′‐GTCTCCTCTGACTTCAACAGCG‐3′, Reverse: 5′‐ACCACCCTGTTGCTGTAGCCAA‐3′. The 2^−ΔΔCT method was used to calculate relative expression levels normalized to GAPDH.

### 2.4 Immunohistochemistry

Formalin‐fixed paraffin‐embedded tissue sections (*n* = 44) were stained with an anti‐ASF1B antibody following standard protocols. Immunoreactive score (IRS) was calculated by multiplying the staining intensity (0–3) and percentage of positive cells (0–4) [20]. The cutoff value to classify high versus low expression was determined as the mean IRS value (7.045) across all tumor tissues.

### 2.5 Statistical Analysis

Statistical analysis was performed using SPSS 27.0. Data were tested for normality using the Shapiro–Wilk test. For normally distributed data, t‐tests were used; for non‐normal data, the Mann–Whitney U test was applied. Categorical variables were compared using the chi‐square test or Fisher's exact test where appropriate.

Classification criteria: HER2 positivity: IHC 3+ or FISH amplification; ER/PR positivity: ≥ 1% nuclear staining; Ki67 positivity: > 20% nuclear staining; Kaplan–Meier curves were compared using the log‐rank test. A *p*‐value < 0.05 was considered statistically significant.

## 3 Result

### 3.1 ASF1B Expression in Breast Cancer

**FIGURE 1:**
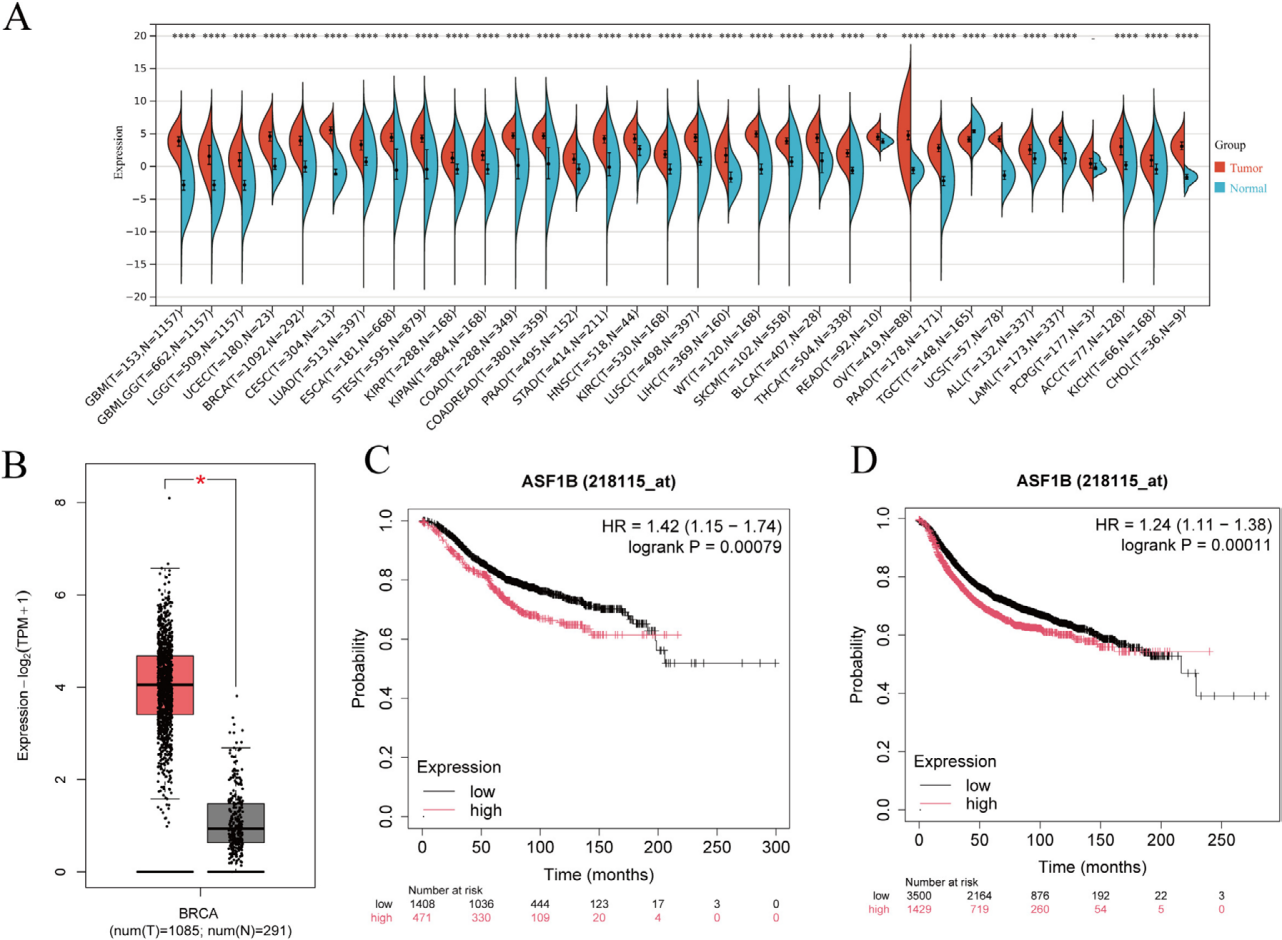
ASF1B expression and prognostic significance in breast cancer. (A) ASF1B expression across various cancer types from the UCSC Xena database. (B) Differential expression of ASF1B in breast cancer versus normal tissues based on GEPIA2. (C) Kaplan–Meier overall survival (OS) analysis comparing ASF1B‐high and ASF1B‐low expression groups. (D) Kaplan–Meier recurrence‐free survival (RFS) analysis stratified by ASF1B expression. High ASF1B expression was significantly associated with worse prognosis (log‐rank test, *p* < 0.001). BRCA, breast cancer; OS, overall survival; RFS, recurrence‐free survival.

ASF1B expression was analyzed in pan‐cancer datasets using the UCSC Xena platform, which revealed widespread overexpression across multiple tumor types. Specifically, ASF1B levels were significantly higher in breast cancer tissues compared to normal controls, as confirmed by the GEPIA2 database (Figure 1A,B; *p* < 0.05).

These findings suggest that ASF1B may play a role in breast tumorigenesis. As a histone chaperone, its upregulation could reflect increased chromatin remodeling activity and cell proliferation capacity in malignant tissues.

### 3.2 Relationship Between ASF1B Expression and Prognosis

Kaplan–Meier analysis showed that breast cancer patients with high ASF1B expression had significantly reduced 10‐year overall survival (OS; HR = 1.42, *p* < 0.001) and recurrence‐free survival (RFS; HR = 1.24, *p* < 0.001), indicating that elevated ASF1B is associated with a worse prognosis (Figure 1C,D).

**FIGURE 2:**
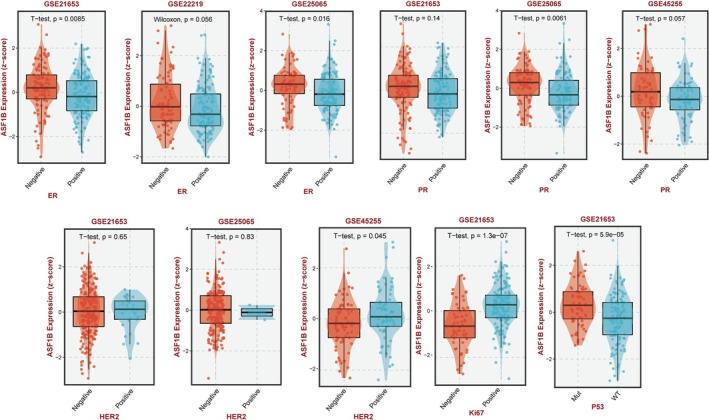
Correlation between ASF1B expression and clinicopathologic markers. ASF1B expression levels stratified by ER, PR, HER2 status, Ki67, and P53 in public datasets (GSE21635, GSE25065, and GSE45255). Higher expression was significantly associated with HER2‐positive, Ki67‐high, and P53‐positive tumors (*p* < 0.05).

Further analysis using the BEST platform across multiple GEO datasets (e.g., GSE21635, GSE25065, and GSE45255) confirmed the consistent overexpression of ASF1B in aggressive molecular subtypes. Notably, higher ASF1B levels were significantly associated with Ki‐67 and P53 positivity, both markers of proliferation and tumor aggressiveness (Figure 2).

**FIGURE 3:**
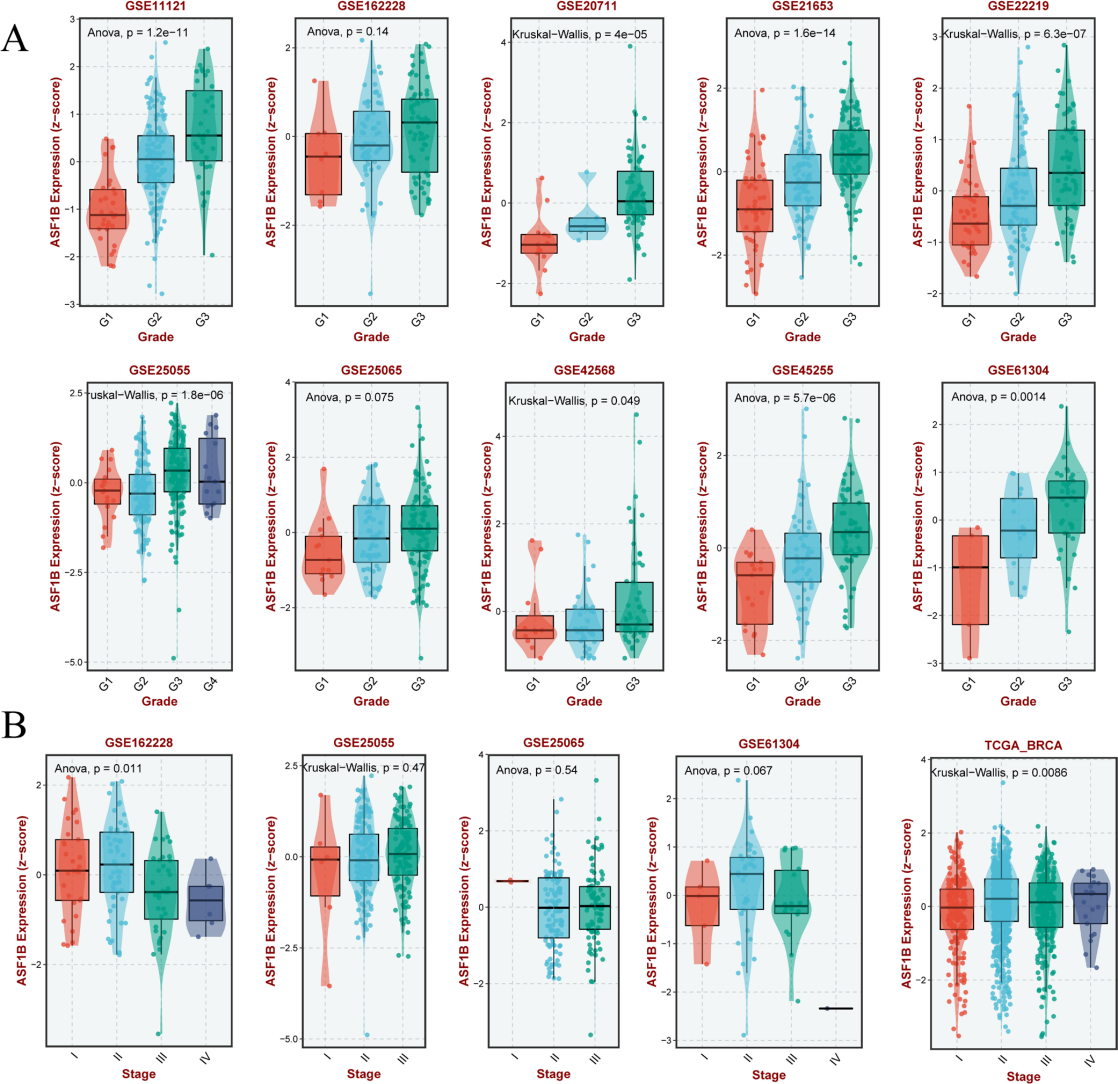
ASF1B expression and tumor grade and stage. (A) Comparison of ASF1B expression across different tumor grades in multiple datasets. (B) ASF1B expression according to clinical stage (I–III). Data sourced from BEST and TCGA platforms.

Figure 3 shows that ASF1B expression is correlated with higher tumor grade and advanced clinical stage in several datasets. These results support a role for ASF1B as a potential prognostic biomarker in breast cancer.

### 3.3 Protein Interaction Network and Enrichment Analysis

**FIGURE 4:**
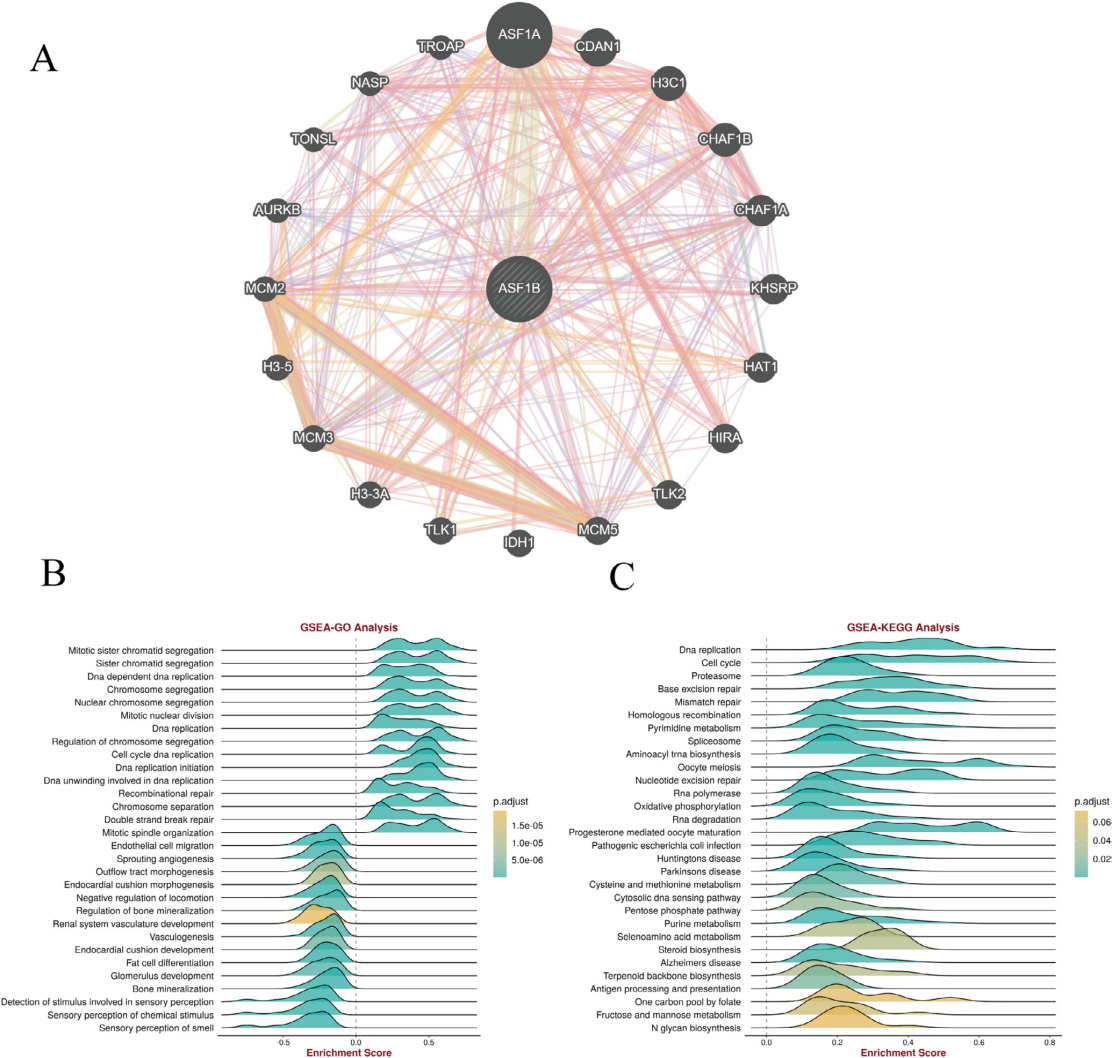
Functional annotation of ASF1B‐associated genes. (A) Gene interaction network constructed using GeneMANIA, identifying co‐expressed genes related to ASF1B. (B) GO enrichment analysis showing involvement in DNA replication and mitosis. (C) KEGG pathway analysis highlighting enrichment in cell cycle and chromatin assembly processes.

Protein interaction analysis via GeneMANIA demonstrated that ASF1B interacts with key regulators of DNA replication and chromatin structure, including ASF1A, CDAN1, and CHAF1B (Figure 4A).

GO and KEGG enrichment analysis indicated that ASF1B‐related genes were enriched in cell cycle progression, mitotic processes, and DNA synthesis pathways (Figure 4B,C). These pathways are frequently dysregulated in rapidly proliferating cancers, further implicating ASF1B in tumor biology.

### 3.4 Immune Microenvironment

**FIGURE 5:**
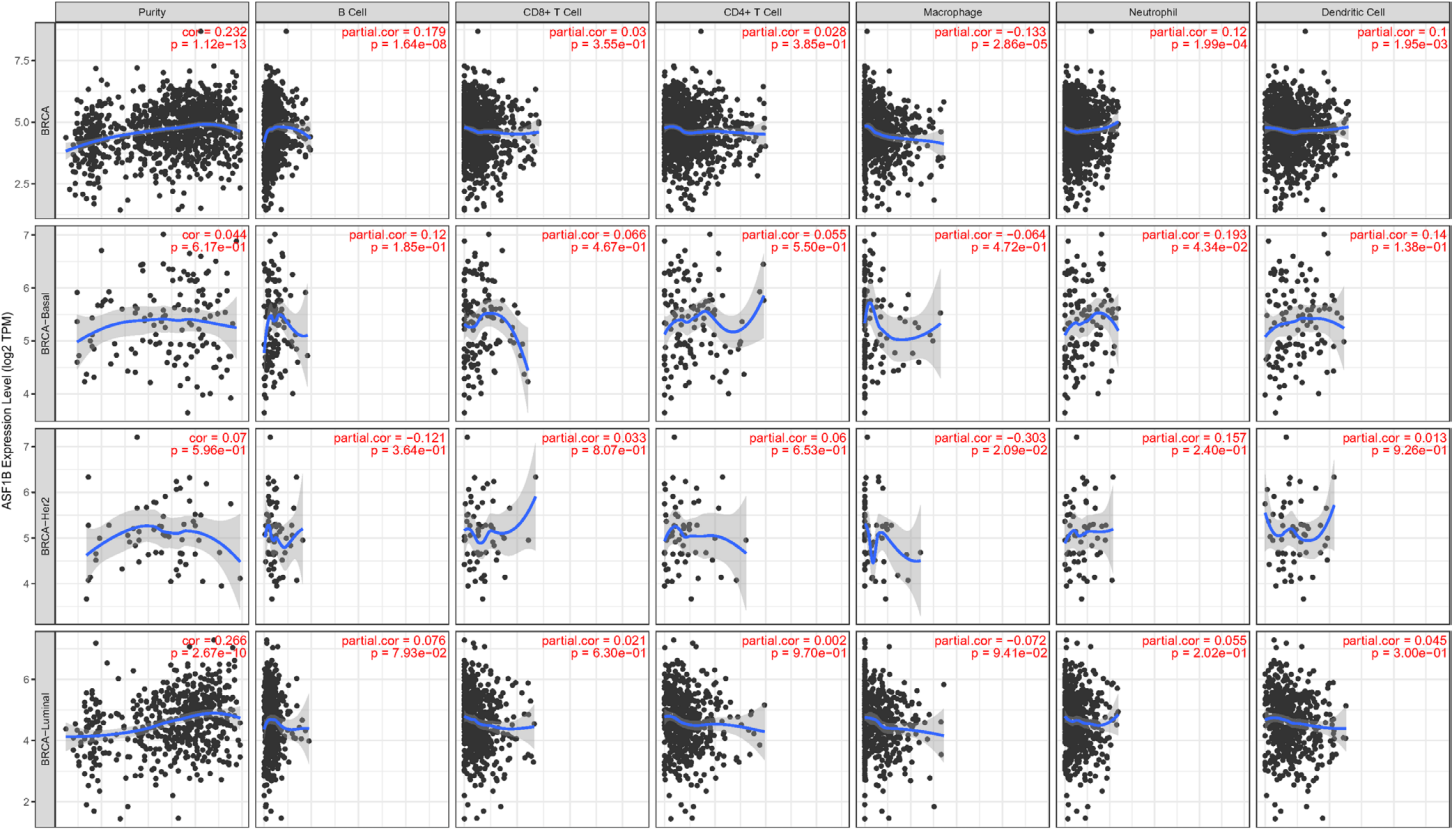
Association between ASF1B expression and immune infiltration. ASF1B expression was positively correlated with the abundance of B cells, dendritic cells, and neutrophils, but negatively correlated with macrophage infiltration (*p* < 0.05). No significant correlations were found with CD4+ or CD8+ T cell populations.

TIMER database analysis revealed that ASF1B expression positively correlated with infiltration of B cells, neutrophils, and dendritic cells and negatively with macrophages (all *p* < 0.05) in breast cancer tissues (Figure 5). However, no significant correlation was observed with CD4+ or CD8+ T cells.

**FIGURE 6:**
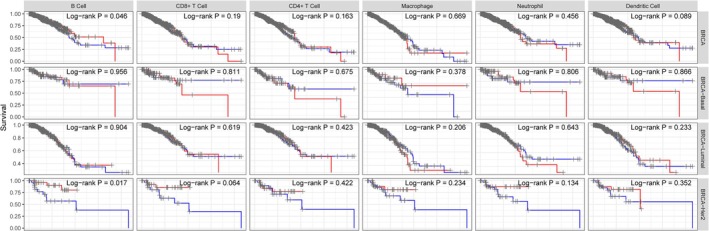
Subtype‐specific immune correlations of ASF1B expression. (A) In HER2‐enriched breast cancer, ASF1B expression negatively correlated with macrophage infiltration (*p* < 0.05). (B) No statistically significant associations were observed in Luminal A or TNBC subtypes.

In HER2‐enriched subtypes, the negative association between ASF1B and macrophages was particularly significant (*p* = 0.021), suggesting subtype‐specific immune modulation. Further subtype analysis showed that ASF1B expression in Luminal A and TNBC subtypes did not significantly correlate with immune cell infiltration (Figure 6).

These results imply that ASF1B may alter immune cell recruitment in a subtype‐dependent manner, possibly affecting immunotherapy responsiveness.

### 3.5 Clinical Sample Analysis and HER2 Association

Among 44 clinical samples analyzed via IHC, high ASF1B expression was significantly associated with HER2 positivity (42.31% vs. 11.11%; *p* = 0.026), as well as with Luminal B molecular subtype (57.69% vs. 33.33%; *p* = 0.03). No significant association was found with age, BMI, ER, PR, or Ki67 (Table 1).

**TABLE 1:** Expression distribution of 44 immunohistochemical samples.

Clinical factor	ASF1B		*t/Z/χ* ^*2*^	*p*
	Low (*n* = 18)	High (*n* = 26)		
Age, Mean±SD	52.33 ± 9.47	54.85 ± 9.26	−0.88	0.386
BMI, kg/m^2^, M (Q_1_, Q_3_)	23.95 (22.53, 26.38)	24.10 (22.38, 26.24)	0.84	0.933
Age at menarche, M (Q_1_, Q_3_)	13.00 (13.00, 13.25)	13.00 (13.00, 14.25)	0.72	0.474
Underlying disease, *n*(%)				
No	11 (61.11)	13 (50.00)	0.53	0.467
Yes	7 (38.89)	13 (50.00)		
Menstrual state, *n*(%)				
Premenopausal	9 (50.00)	17 (68.00)	1.42	0.234
Postmenopausal	9 (50.00)	8 (32.00)		
Family history, *n*(%)				
No	18 (100.00)	24 (92.31)	1.45	0.228
Yes	0 (0.00)	2 (7.69)		
ER, *n*(%)				
Negitive	2 (11.11)	7 (26.92)	1.63	0.201
Positive	16 (88.89)	19 (73.08)		
PR, *n*(%)				
Negative	4 (22.22)	10 (38.46)	1.29	0.256
Positive	14 (77.78)	16 (61.54)		
Ki67, *n*(%)				
Negative	9 (50.00)	9 (34.62)	1.04	0.307
Positive	9 (50.00)	17 (65.38)		
Her‐2, *n*(%)				
Negative	16 (88.89)	15 (57.69)	4.97	0.026
Positive	2 (11.11)	11 (42.31)		
淋巴结状, *n*(%)				
Negative	8 (44.44)	10 (38.46)	0.16	0.691
Positive	10 (55.56)	16 (61.54)		
T, *n*(%)				
1	8 (44.44)	7 (26.92)	1.96	0.375
2	10 (55.56)	18 (69.23)		
3	0 (0.00)	1 (3.85)		
N, *n*(%)				
0	8 (44.44)	10 (38.46)	2.46	0.482
1	6 (33.33)	11 (42.31)		
2	4 (22.22)	3 (11.54)		
3	0 (0.00)	2 (7.69)		
Molecular typing, *n*(%)				
Her‐2 overexpression	0 (0.00)	3 (11.54)	8.94	0.03
Luminal A	10 (55.56)	4 (15.38)		
Luminal B	6 (33.33)	15 (57.69)		
Triple‐negative	2 (11.11)	4 (15.38)		
Stage, *n*(%)				
I	4 (22.22)	1 (3.85)	6.27	0.18
IIA	6 (33.33)	10 (38.46)		
IIB	4 (22.22)	10 (38.46)		
IIIA	4 (22.22)	3 (11.54)		
IIIC	0 (0.00)	2 (7.69)		
Pathological type, *n*(%)				
Invasive ductal carcinoma	15 (83.33)	23 (88.46)	6.44	0.265
Mucoepidermoid carcinoma	2 (11.11)	0 (0.00)		
Ductal carcinoma in situ	0 (0.00)	1 (3.85)		
Secretory carcinoma of breast	1 (5.56)	0 (0.00)		
Apocrine carcinoma	0 (0.00)	1 (3.85)		
Invasive lobular carcinoma	0 (0.00)	1 (3.85)		

*Note:* The correlation between clinical characteristics and ASF1B gene expression was explored in immunohistochemistry samples (44 cases). The analysis included age, body mass index (BMI), age of menarche, underlying disease, menopause status, family history, ER, PR, Ki67, Her‐2, lymph node status, T and N phases, molecular typing, pathological staging, and pathological diagnosis.

These findings suggest that ASF1B overexpression may be particularly relevant in HER2‐positive and Luminal B breast cancers. This is consistent with the literature indicating that HER2 pathway activation is often accompanied by chromatin remodeling and increased proliferation.

### 3.6 qPCR and IHC Validation

**FIGURE 7:**
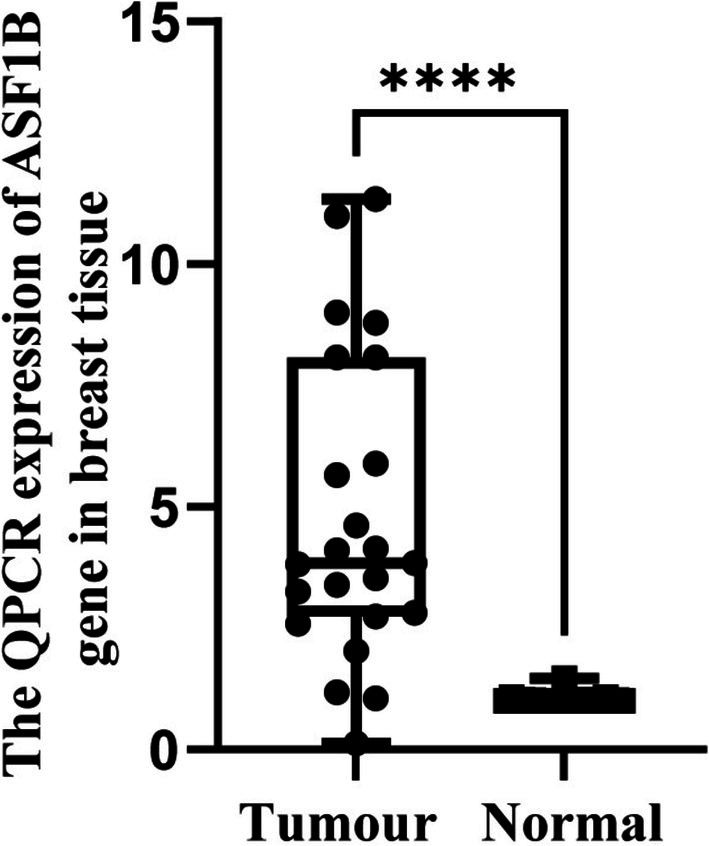
Validation of ASF1B mRNA expression by qPCR. Quantitative PCR analysis of 23 paired breast tumor and adjacent normal tissues revealed significantly higher ASF1B expression in tumors (*p* < 0.001, paired *t*‐test). **** means *p* < 0.0001.

**FIGURE 8:**
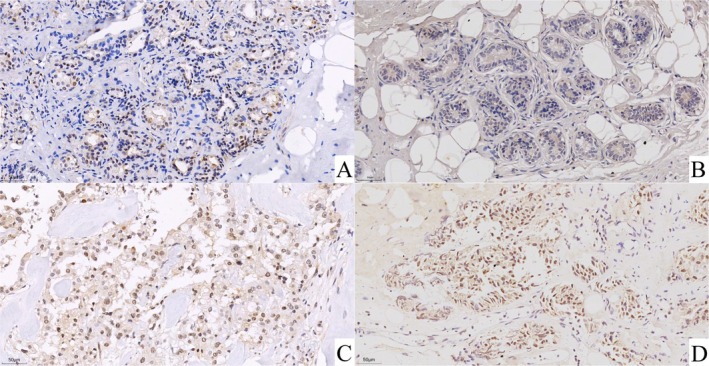
Representative IHC staining for ASF1B in breast cancer. Stronger nuclear and cytoplasmic ASF1B staining was observed in breast tumor tissues compared to adjacent non‐tumorous tissues. Magnification: ×400.

**FIGURE 9:**
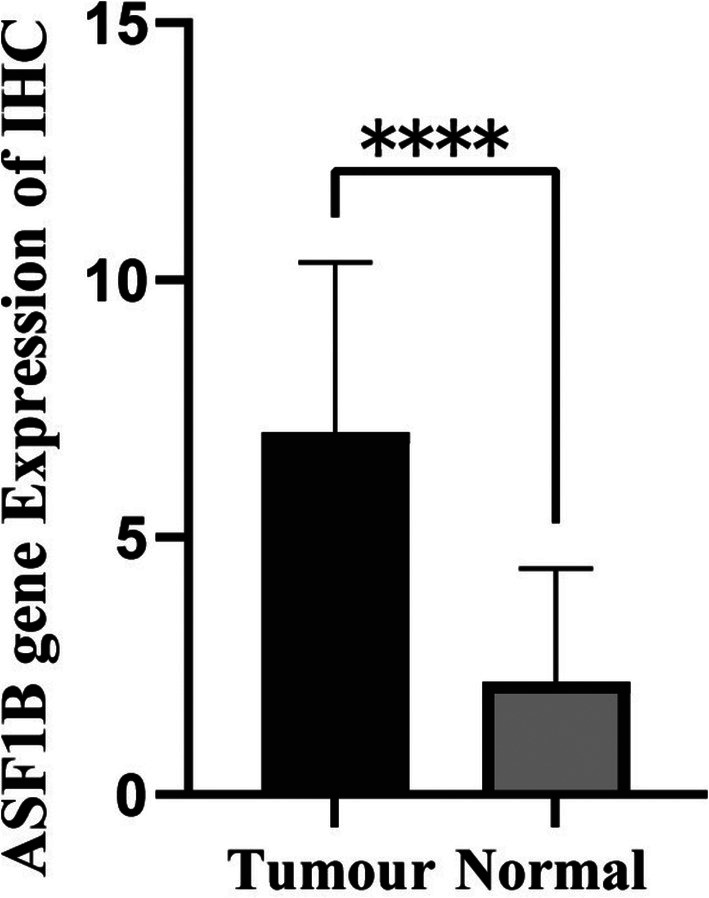
Quantification of ASF1B expression via immunohistochemistry. IRS scores of ASF1B IHC staining in 44 breast cancer cases showed significantly higher values in tumor tissues compared to normal controls (*p* < 0.001, Student's *t*‐test). **** means *p* < 0.0001.

In clinical tissue samples, ASF1B mRNA levels were significantly higher in tumor tissues compared to adjacent non‐cancerous tissues (*p* < 0.001, Figure 7). IHC staining showed stronger ASF1B protein expression in tumor sections, confirmed by semi‐quantitative IRS analysis (*p* < 0.001, Figures 8 and 9).

These experimental results corroborate the bioinformatics findings and support the utility of ASF1B as a diagnostic and prognostic marker.

## 4 Discussion

In this study, we conducted a comprehensive bioinformatics and clinical validation analysis to investigate the expression pattern, clinical relevance, and immunological associations of anti‐silencing function protein 1B (ASF1B) in breast cancer. Our findings demonstrate that ASF1B is significantly overexpressed in breast cancer tissues compared to normal controls, and that its elevated expression is associated with poor prognosis, aggressive molecular features, and altered immune cell infiltration.

ASF1B, as a histone H3–H4 chaperone, plays a key role in chromatin assembly during DNA replication and repair. Its overexpression in breast cancer may promote tumor cell proliferation by facilitating rapid cell cycle progression and chromatin accessibility [4]. Prior studies in gastric, lung, and cervical cancers have suggested that ASF1B modulates cell cycle regulators such as Cyclin E1, CDK2, and MYC, consistent with our enrichment results showing associations with DNA replication and mitotic pathways [21].

Of particular interest is the significant correlation between high ASF1B expression and HER2‐positive breast cancer. HER2 amplification is known to drive aggressive tumor phenotypes via PI3K/AKT and MAPK signaling cascades [22]. Our results suggest that ASF1B might interact with these proliferative pathways by enhancing chromatin remodeling at key promoter regions, thereby contributing to HER2‐driven oncogenesis. Although further mechanistic studies are needed, ASF1B may represent a downstream effector or co‐regulator in HER2‐mediated tumorigenesis.

In the immune context, ASF1B expression was significantly associated with increased infiltration of B cells, dendritic cells, and neutrophils, but decreased macrophage infiltration. These correlations suggest a potential immunomodulatory function. For example, high ASF1B expression may alter cytokine or chemokine production (e.g., IL‐6 and TNF‐α), thereby shaping the immune milieu. Interestingly, in HER2+ tumors, ASF1B was inversely correlated with macrophage presence—possibly indicating suppression of M2‐type tumor‐promoting macrophages. Future studies should explore whether ASF1B regulates macrophage polarization (e.g., M1 vs. M2) through epigenetic control of transcription factors such as STAT3 and NF‐κB [23].

Our clinical analysis further confirmed that ASF1B expression is positively correlated with HER2 status and Luminal B subtype, both known to be associated with higher proliferation indices. These findings support ASF1B's potential as a biomarker for identifying high‐risk patients who may benefit from intensified treatment or novel targeted therapies.

Although our results are robust and consistent across datasets, the study has limitations. First, the sample size for experimental validation (*n* = 67) is relatively small, which may limit the generalizability of the clinical associations. Second, due to limited resources, we did not perform functional experiments (e.g., siRNA knockdown and invasion/migration assays) to confirm the biological role of ASF1B. Future research should aim to validate our hypotheses in vitro and in vivo, including exploring ASF1B's impact on treatment resistance and immunotherapy response.

Despite these limitations, our study is, to our knowledge, the first to integrate large‐scale database analysis with clinical sample validation to characterize ASF1B in breast cancer, especially its link to HER2 biology and immune infiltration. These findings highlight ASF1B as a promising diagnostic and prognostic biomarker and suggest it may be a potential therapeutic target, particularly in HER2‐enriched and immune‐infiltrated tumors.

In conclusion, ASF1B is significantly upregulated in breast cancer and is associated with poor clinical outcomes and distinct immune signatures. Its expression correlates with HER2 status and immune cell distribution, suggesting both oncogenic and immunomodulatory roles. These results provide a foundation for future research into ASF1B‐targeted therapies and underscore its potential value in personalized breast cancer management.

## Author Contributions

**Wenhao Xing:** data curation, writing – original draft, visualization, validation, software. **Meng Deng:** investigation, methodology, resources. **Wendong Wang:** writing – review and editing, supervision. **Yueqi Liu:** data curation. **Xuefang Mi:** formal analysis. **Huixia Li:** visualization. **Xin Ge:** resources, funding acquisition, supervision, writing – review and editing.

## Ethics Statement

The study plan has been approved by the Ethics Review Committee of the First Affiliated Hospital of Zhengzhou University (Program No.: 2024‐KY‐2094‐002), Zhengzhou, China, and all patients have provided written informed consent. All procedures follow the guiding principles of the Declaration of Helsinki.

## Conflicts of Interest

The authors declare no conflicts of interest.

## Data Availability

The data that support the findings of this study are openly available in NCBI at https://www.ncbi.nlm.nih.gov/geo/.
